# Learning new words from an interactive electronic storybook intervention

**DOI:** 10.4102/sajcd.v65i1.601

**Published:** 2018-09-13

**Authors:** Daleen Klop, Laurette Marais, Amanda Msindwana, Febe de Wet

**Affiliations:** 1 Division of Speech-Language and Hearing Therapy, Stellenbosch University, South Africa; 2 Human Language Technology Research Group, Meraka Institute, Council for Scientific and Industrial Research (CSIR), Pretoria, South Africa

## Abstract

**Background:**

Children who enter school with limited vocabulary knowledge are at risk for reading failure. This study investigated the efficacy of an interactive e-book, implemented as a mobile application, to facilitate vocabulary learning in Grade 1 isiXhosa-speaking children (*n* = 65).

**Objective:**

The purpose was to measure if an e-book intervention, specifically developed for use in the South African context, could facilitate the acquisition and retention of new words at different levels of lexical representation.

**Method:**

A randomised pre-test and/or post-test between-subject design was used where an experimental group that received the e-book intervention was compared to a control group before the control group received a delayed intervention. Follow-up testing was performed to measure retention of the new vocabulary after eight weeks. Mixed-model repeated-measure Analysis of Variance (ANOVAs) were used to determine differences between the participants in the experimental and control groups.

**Results:**

The short-term e-book intervention not only facilitated fast-mapping of new words but enabled participants to develop more robust lexical representations of the newly acquired words. Follow-up assessment showed that they retained their newly acquired word knowledge.

**Conclusion:**

Multimedia technology can be used to provide explicit and embedded vocabulary training to young children at risk for academic failure. These findings are particularly relevant for South African environments where there is limited parental support and lack of educational resources to promote vocabulary learning in young children.

## Introduction

South Africa is faced with an ongoing crisis in literacy and reading, as our children perform poorly when compared to international standards. Many South African children are at risk for scholastic failure because of the pervasive and persistent inequalities in educational resources, risk factors associated with poverty and limited parental support, and inadequate instruction in overcrowded classrooms.

The 2016 Progress in International Reading Literacy Study (PIRLS) showed that of the 12 810 Grade 4 children who were assessed in 293 schools across South Africa, 78% did not reach the low international benchmark for reading. They could not perform basic reading skills such as reading for meaning, locating and retrieving explicitly stated information or make inferences about events or provide reasons for actions (Howie et al., 2017).

Vocabulary, the knowledge of word meanings, is a powerful predictor of reading comprehension. Children with a limited vocabulary struggle with reading comprehension because they lack sufficient depth of word knowledge to process the meaning of more complex words or words in different morphosyntactic forms. They have less efficient lexical processing skills, are less likely to learn new words from incidental or embedded exposure to words and need repeated and explicit instruction to acquire deeper word knowledge (Coyne et al., 2009).

In South Africa, Wilsenach (2015) found that receptive vocabulary size in emergent bilingual Northern Sotho–English children predicted the development of their early literacy skills. Children with a limited vocabulary not only lag behind their peers at school entry, but the gap widens as they progress through school. Pretorius and Stoffelsma (2017) found that a group English First Additional Language learners in the Eastern Cape only knew 27% of the most frequent words in their textbooks by the end of Grade 3.

Research has shown that children at risk for literacy failure need not only code-based reading instruction but also interventions to improve language and vocabulary skills (Biemiller & Boote, 2006; Coyne et al., 2009). According to Nation (2013) the most effective ways to maximise vocabulary learning are to ensure that children do large amounts of reading, using graded material at the appropriate developmental level, repeated exposure to targeted vocabulary and deliberate learning through linked activities such as reading with exercises and computer-based activities.

There is therefore a growing focus on the development of programmes and procedures to improve and even accelerate vocabulary growth in children at risk for literacy failure. One such option is the development of multimedia interventions to support the language and literacy learning skills of children growing up in lower socio-economic status (SES) home and school environments. In this article we report the results of using an interactive electronic book (e-book) to facilitate vocabulary learning in children from disadvantaged backgrounds.

The use of technology is a promising solution to support the development of literacy and to compensate for the language gap between linguistically advantaged and disadvantaged children (Bus & Neuman, 2009). One of the recommendations in response to the 2016 PIRLS results was that effective and sustainable access to information and communications technology (ICT) in South African primary schools should be increased (Howie et al., 2017). For our study we developed an original interactive e-book, implemented as a mobile application.

The purpose of our study was to measure the efficacy of an e-book, specifically developed for use in the South African context, to facilitate the learning of new words in children at risk for scholastic failure. The results of an earlier study reported in De Wet, Pretorius and Klop (2015) showed that Afrikaans-speaking Grade 1 participants (*n* = 28) from disadvantaged backgrounds acquired and retained new words after short-term exposure to an e-book intervention. This follow-up study was conducted to determine if similar changes would emerge in a different test population, namely isiXhosa-speaking children in a lower SES rural environment in the Eastern Cape.

The study was guided by the following research question: what is the effect of an interactive e-book intervention on vocabulary learning of a group of isiXhosa-speaking mainstream Grade 1 children?

The specific research questions were as follows:

- What are the differences between the participants’ knowledge of 15 target words before and after the intervention in terms of the following levels of word learning:
  ■ Receptive or partial knowledge?
  ■ Contextual or deeper knowledge?
  ■ In-depth or full knowledge?
- Will they retain their knowledge of new words at the different levels of learning?

The hypothesis, based on the results of De Wet et al. (2015), was that participants would acquire and retain new words from their exposure to the e-book intervention.

## Method

### Study design

A randomised pre-test and/or post-test between-subject design was used, where an experimental group that received the intervention was compared to a control group before the control group received a delayed intervention.

### Participants

Participants were recruited from Grade 1 classes in two isiXhosa-medium schools in the district of Ntsika Yethu-Cofimvaba, in the Eastern Cape. The community comprise mainly subsistence farmers, and many children are cared for by grandparents while their parents are migrant workers in urban areas. Both schools had only one Grade 1 class. As a result, the classrooms were overcrowded, with 51 children in one class and 91 children in the other class. The quality of teaching and learning in both schools was further compromised by a lack of learning resources available in the classrooms and children’s poor school attendance.

For inclusion in the study, children had to pass otoscopic examinations, pure tone hearing and optometric screening tests to ensure that all participants had normal hearing and vision. Children were assessed individually by two researchers who were fluent isiXhosa speakers, in a quiet room in the school. Their non-verbal skills were assessed by means of the Test of Nonverbal Intelligence, Fourth Edition (TONI-4) (Brown, Sherbenou & Johnsen, 2010), a language-free measure of cognitive ability. The purpose of this measure was to enable comparisons between participant groups in terms of their cognitive ability. Participants’ TONI-4 index scores ranged from 77 to 106 (scores below 90 indicate below-average performance). There are no standardised language tests available for isiXhosa-speaking children and participants’ language skills could therefore not be assessed formally. Inclusion criteria stipulated that the children had to be isiXhosa first-language speakers, aged between 6 and 8 years. Children older than 8 years, and children who failed the hearing and visual screening tests, were excluded from the study.

After matching for gender, participants who complied with the selection criteria were randomly assigned to the experimental and control groups. Because of poor school attendance in general, many children were absent during the assessment and intervention period. Only 65 of the 140 children who consented to participation fulfilled all the selection criteria and completed all the phases of the study. The experimental group (*n* = 33) comprised 16 boys and 17 girls, and the control group (*n* = 32) comprised 13 boys and 19 girls; their average age was 6 years and 7 months. The groups were comparable in terms of age and non-verbal intelligence. One-way Analysis of Variance (ANOVA) showed no differences between the groups for TONI-4 index scores, *F*(1, 63) = 3.1670, *p* = 0.08.

## Materials and instrumentation

### Storybook

**FIGURE 1:**
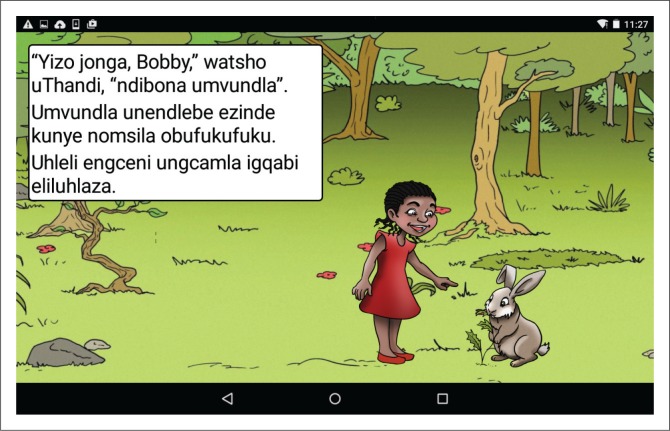
Screenshot of story on tablet.

An original story with learning activities was created by the first author for the study reported in De Wet et al. (2015). It was translated from Afrikaans to isiXhosa by the third author, a first-language isiXhosa speaker who is fluent in Afrikaans. A professional artist illustrated the story under the guidance of the first author to ensure that each picture portrayed the target words and storyline. Adjustments to the story and pictorial content were made to the Afrikaans story to accommodate the linguistic differences between Afrikaans and isiXhosa. For instance, for the isiXhosa version the illustrator altered the rabbit’s tail to depict the target word *fukufuku* (a word to describe the texture of a dandelion seed) more accurately. Figure 1 presents a screenshot from the e-book.

### Vocabulary content

**FIGURE 2:**
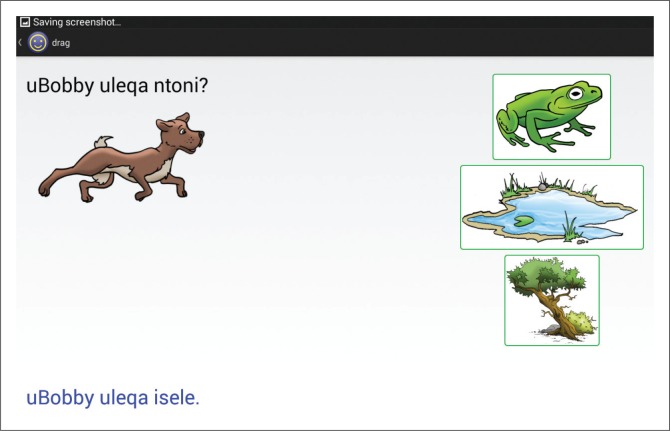
Example of an interactive activity.

The e-book contained 15 target words (5 nouns, 4 verbs, 1 adverb and 5 adjectives) that were embedded in the story context (see Box 1). The 15 words were selected to represented Tier 2 words, that is, words that are used frequently by adults and in books but are unlikely to be familiar to young children, although they would be able to understand them in context (Beck & McKeown, 2007). Figure 2 illustrates an interactive activity.

BOX 1 Fifteen target words

**Table:** 

Target words:
1. *ngcamla* – taste (verb)
2. *mtybilizi* – slimy/slippery (adjective)
3. *umngxumo* – hole (noun)
4. *ephothiweyo* – woven (adjective)
5. *khuselekile* – safe/protected (adjective)
6. *ntupha* – paw (noun)
7. *khatha* – dive in/enter in (verb)
8. *fukufuku* – fluffy (adjective)
9. *ichibi* – dam/pool (noun)
10. *ihlathi* – forest (noun)
11. *zimela* – hide (verb)
12. *acwengileyo* – clear (adjective)
13. *ulwamvila* – bee sting (thorn) (noun)
14. *ngcileza* – hop/skip (verb)
15. *krekretha* – bite hard (adverb)

## Application development

The application included three subcomponents, namely the home page with activity choices, such as activities of interaction with the e-book content and thirdly multichoice-style quizzes with questions that were answered by clicking on or dragging an object to the correct answer. The home page of the application provided buttons for accessing six different activities. The activities included reading through the e-book content and interacting with it, as well as multichoice-style activities for answering questions about the e-book content.

The e-book was implemented as a mobile application, specifically for Android tablets. It used a text-to-speech (TTS) engine to generate the audio version of the text displayed on the screen. The application used the Qfrency^1^ TTS3 isiXhosa voice to convert TTS. Qfrency TTS is a speech synthesis system that was developed specifically for South Africa’s official languages. Technical details on application implementation are provided in De Wet et al. (2015).

Children first listened to the story read by a voice while observing the pictures on the tablet. The interactive activities, based on the story content, were added to facilitate participants’ active engagement with the target words through manipulation and discrimination tasks. The aim was to provide more opportunities to interact with the target words to consolidate and reinforce comprehension and to enable deeper word learning. The frequency of the target words was controlled for the number of times each word occurred in the story text and the learning activities to make comparisons between words possible.

## Procedures

### Pre-testing

**FIGURE 3:**
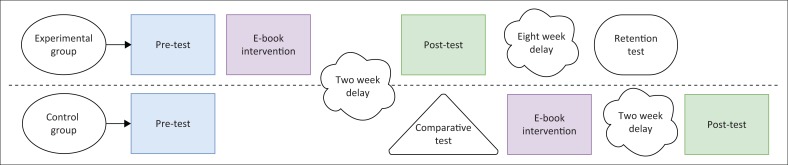
Assessment and intervention schedule.

Measurement tasks were developed by the researchers to test participants’ knowledge of the 15 target words at three task levels prior to the e-book intervention. The assessments were based on the protocol that was piloted in the De Wet et al. (2015) study and were deemed valid by the researchers to assess the three levels of vocabulary learning. Training items were provided to make sure that the children understood the tasks and instructions. The assessment protocol is shown in Appendix 1. The study timeline is shown in Figure 3.

## Vocabulary measures

### Receptive vocabulary knowledge task (partial knowledge)

This task entailed matching a word with its pictorial referent out of three distractors, for example, ‘show me woven (*ephothiweyo*)’. This activity required only partial knowledge of a word, and a correct response did not necessarily indicate full understanding of the word. Each correct answer received one point.

### Sentence completion task (contextual and/or deeper knowledge)

This measure assessed expressive knowledge of the target words within the context of a sentence, for example, ‘the rabbit’s tail is … ? (fluffy)’. The cue sentences were novel sentences and not taken verbatim from the story or interactive activities to prevent responses that were just repetition from memory. Each correct answer received one point.

### Word definition task (in-depth and/or full knowledge)

This measure assessed how well children understood the target words and whether they were able to express their understanding in a meaningful explanation, for example, ‘what do you think *woven* means?’ No explicit definitions or word explanations were provided in the story or interactive activities. Participants’ responses were coded on a four-point scale: 0 points for no or incorrect responses, one point for a response that used the word appropriately but without an explanation or elaboration, for example, ‘the nest is woven’, two points for a response that indicated attempts to explain or elaborate, for example, ‘woven is like the bird’s nest’, three points for a definition or description that indicated full understanding of the word, for example, ‘woven means the bird used grass to make his nest’.

## E-book intervention

The experimental group (*n* = 33) received the e-book intervention in groups of 3–4 children, three times per week in 20-min sessions, for a period of 2 weeks, that is, 6 sessions (2 h) in total. Two weeks after the programme was completed, the vocabulary assessment battery was repeated on both groups. The control group (*n* = 32) then received the e-book intervention programme in the same way as the experimental group.

The intervention sessions were supervised and directed by one of the researchers, who helped the children to become familiar with the tablet and to access the interactive e-book. Children listened to the e-book through headphones to maximise the sound quality of the application and to avoid distractions. They engaged independently with the story and activities, following the verbal instructions that were part of the e-book. All the children had previous exposure to tablets and were familiar with interacting with educational material on tablets.

## Post- and follow-up testing

To minimise memory effects, post-tests to assess and compare both groups’ knowledge of the target words took place two weeks after the experimental group completed the intervention. After a period of 8 weeks, the assessments were repeated on the experimental group to determine their retention of the newly acquired words. The control group’s post-tests took place 2 weeks after they completed the intervention to assess their word learning. The control group was not assessed again for retention, as it was felt that a fourth assessment with the same battery could have elicited training effects. To minimise training effects between the tasks, participants were always tested first with the definition task, then with the sentence completion task and lastly with the receptive task.

## Data coding and analysis

The results of the screening and the pre- and post-intervention assessments were manually coded and analysed by the third author. To ensure inter-rater scoring reliability, the word definition task was scored independently by another person who was an isiXhosa first-language speaker and unaware of participants’ group assignment. Point-by-point agreement, calculated by comparing the total number of agreements with the agreements plus disagreements, was 98%. A software package, Statistica 12, was used for the statistical analyses. To examine the differences between the participants in the experimental and control groups, mixed-model repeated-measure ANOVAs were used. A 5% significance level (*p* < 0.05) was used as the guideline for determining significant effect.

## Ethical consideration

The study formed part of a project approved by the Council for Scientific and Industrial Research (CSIR) Ethics Committee (62/2013). Formal written consent was obtained from the school principals, parents or caregivers and verbal assent from the participants prior to the study.

## Results

The main aim of the study was to determine the efficacy of an interactive e-book to facilitate vocabulary learning. As shown in Table 1, both groups had similar pre-test scores, but the experimental group performed significantly better than the control group when assessed after the intervention. The control group’s post-test scores showed that their scores improved in a similar way following the intervention. The experimental group’s follow-up results, 8 weeks after completion of the intervention programmes, indicated that they had retained these gains.

**TABLE 1:** Means (and standard deviation) for receptive vocabulary, sentence completion and word definition scores for participants in the experimental (*n* = 33) and control (*n* = 32) groups for the pre-, post- and follow-up assessments.

Assessment task	Pre-test					Post-test								Follow-up	
Variables	Exp. group		Control group		Exp. group		Control group		Exp. group		Control group	
	*n*	SD	*n*	SD	*n*	SD	*n*	SD	*n*	SD	*n*	SD
Receptive vocabulary (max score 15)	6.6	2.5	6.4	2.1	12.2	2.4	7.2	2.3	12.1	2.5	11.7	3.3
Sentence completion (max score 15)	0.5	0.6	0.6	0.7	4.4	2.1	1.2	1	4.7	2.5	4.2	2.3
Word definitions (max score 45)	9.1	3.1	9	4.8	17	5.2	10.7	4.6	21	5.3	21.8	7.7

Exp, experimental; SD, standard deviation.

### Receptive vocabulary

**FIGURE 4:**
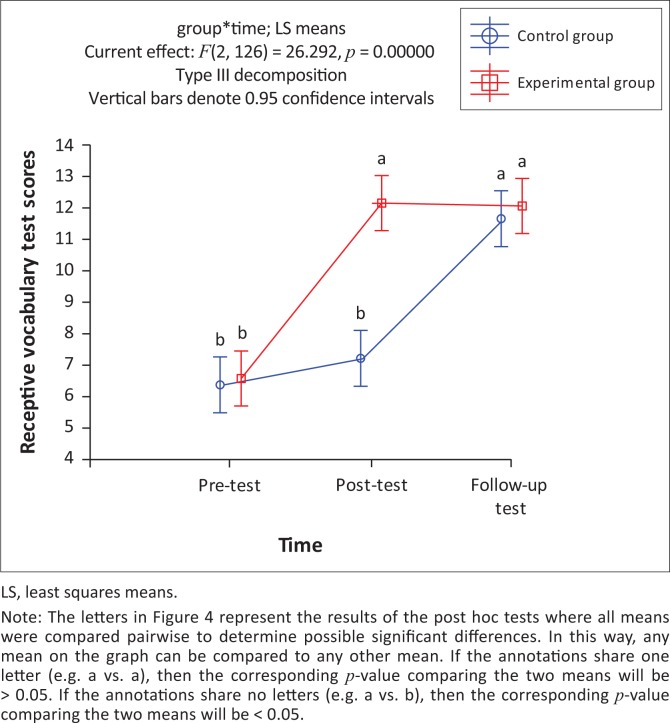
Receptive vocabulary: Mean scores of participant groups for pre-, post- and follow-up assessment.

A significant interaction between group and time was found, *F*(2, 126) = 26.29, *p* < 0.05. As shown in Figure 4, most of the participants demonstrated at least partial word learning of most of the 15 target words as a result of their exposure to the words in the e-book intervention.

### Sentence completion

**FIGURE 5:**
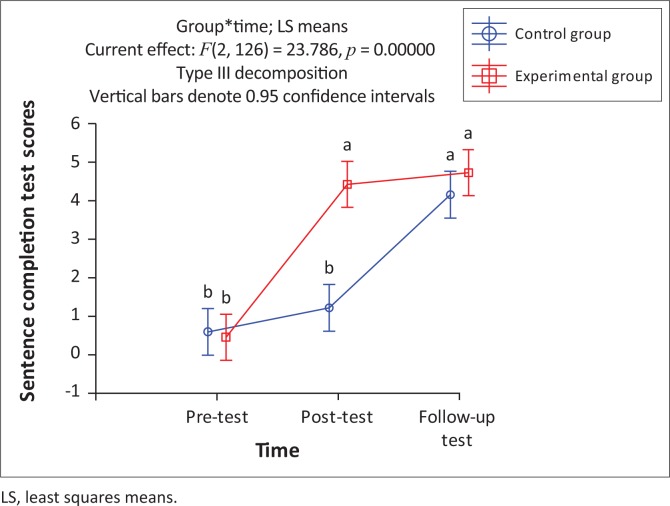
Expressive vocabulary: Mean scores of participant groups for pre-, post- and follow-up assessment of the sentence completion task.

A significant interaction between group and time was found, *F* (2, 126) = 23.79, *p* < 0.05. As shown in Figure 5, few of the participants could use any of the 15 target words to complete the cue sentence prior to the intervention. This result was expected, as only the target word was accepted as correct, even if another word was used appropriately to complete the sentence. Results of the post- and follow-up tests demonstrated that modest gains in expressive word learning took place and that most participants could use some target words in a meaningful sentence context.

### Word definitions

**FIGURE 6:**
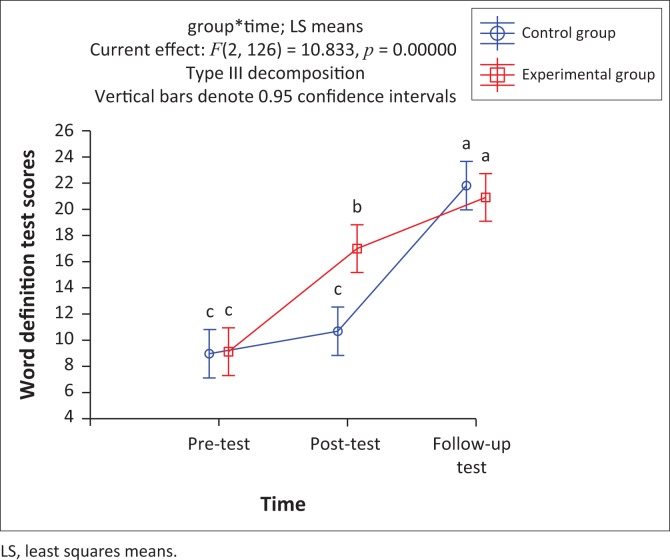
Mean scores of participant groups for pre-, post- and follow-up assessment of the word definition task.

This subtest assessed the children’s ability to provide definitions of the 15 target words and to assess the depth of their word learning. As shown in Figure 6, significant interaction between group and time was found, *F*(2, 126) = 10.83, *p* < 0.05. The post- and follow-up tests demonstrated therefore that most participants could provide some form of expressive explanation or interpretation of the target words, indicating that they acquired not only partial knowledge of word meanings but also deeper and more complete understanding of the target words.

## Discussion

Our intervention aimed not only to facilitate fast-mapping of new words but to enable participants to develop more robust lexical representations of the newly acquired words. The results showed that all the participants acquired new Tier 2 level words at different levels of semantic representation. Moreover, in contrast with the findings of other studies (e.g. Coyne et al., 2009), their word learning over time was robust and they retained not only their gains in partial word knowledge but also at deeper levels of word understanding and lexical representation. This is particularly significant for such a short-term intervention; children were only exposed to the 15 target words for 2 h in total over a period of 2 weeks. It should be acknowledged that learning effects could have occurred during the post-intervention assessments, but the stability of vocabulary scores even after 8 weeks is encouraging.

## Limitations

This study only used one e-book that was created specifically for the project. It can, therefore, not be inferred that similar learning effects would be observed for all e-books. Most commercially available e-books are created for entertainment purposes and may be less effective in facilitating word learning than an e-book that was created with vocabulary intervention goals in mind. Further research to investigate differences in learning effects for e-books, with and without additional training activities, could inform developers and consumers about the most effective ways to use e-books for word learning.

## Clinical implications

One of the main benefits of e-book interventions is that children can engage with the content and activities independently with limited adult mediation. In an environment where there is little parental support at home and too many children in school classrooms, opportunities for shared storybook reading are often inadequate. E-books can, therefore, fill an important gap by enabling children from these environments to direct their own learning experiences and benefit from regular exposure to stories. E-books, in contrast with conventional shared reading, can also offer additional learning opportunities through interactive activities to reinforce and consolidate learning of new words.

## Conclusion

The short-term e-book intervention facilitated the acquisition of new words in a group of isiXhosa-speaking Grade 1 children at risk for scholastic failure. The results confirmed the findings of the earlier study in an Afrikaans-speaking population (De Wet et al., 2015). It can therefore be concluded that this e-book intervention is an effective tool to facilitate vocabulary learning, even across linguistic populations.

## Footnotes

1 http://www.qfrency.com/

## Appendix 1

### Assessment protocol

#### Icandelo A:

Isigama esichazwayo

**ITAFILE 1-A1:** Inkcazo yamagama - Isivakalasi esitshiwo kuqala: Uthetha/Ithetha ntoni … ?

Uvavanyo	Impendulo yomntwana	Isikali samanqaku			
1. ukungcamla		0	1	2	3
2. emtybilizi		0	1	2	3
3. emngxunyeni		0	1	2	3
4. ephothiweyo		0	1	2	3
5. ekhuselekile		0	1	2	3
6. intupha		0	1	2	3
7. ithi khatha		0	1	2	3
8. obufukufuku		0	1	2	3
9. ichibi		0	1	2	3
10. ihlathi		0	1	2	3
11. ukuzimela		0	1	2	3
12. acwengileyo		0	1	2	3
13. ulwamvila		0	1	2	3
14. ngcileza		0	1	2	3
15. ukukrekretha		0	1	2	3

**ITAFILE 2-A1:** Izivakalisi ezingaphelelanga

Uvavanyo	Impendulo	Elungileyo/Engalunganga
1. Umvundla uxakekile negqabi uyali ……… (ngcamla)		
2. Isikhumba sesele si ………. (mtyibilizi)		
3. Impuku ihlala kumthi phakathi ……… (emngxunyeni)		
4. Indlwana yakhiwe ngengca ……… (ephothiweyo)		
5. Isele aloyiki kuba …….. (likhuselekile)		
6. uBobby udlala ngee …. (ntupha)		
7. Impuku ingena emngxunyeni ithi ….. (khatha)		
8. Umsila womvundla ……….. (ubufukufuku)		
9. Amanzi afumaneka ………. (echibini)		
10. Imithi ikhula …….. (ehlathini)		
11. Ihobohobo lingene kwindlwana yalo ……. (lazimela)		
12. Amanzi edamini …….. (acwengile)		
13. Inyosi ihlaba ngo ……. (lwamvila)		
14. Umvundla uyewaya apha naphaya …… (engcileza)		
15. Impuku ixakekile nentongomane iyali …… (krekretha)		
**Isambuku sempendulo ezichaniweyo**		

#### Icandelo B:

Isivakalasi esitshiwo kuqala: Khomba (yolatha) ngomnwe wakho…

**ITAFILE 3-A1:** Isigama esiqondwayo - Isivakalasi esitshiwo kuqala: *Khomba* (*yolatha*) *ngomnwe wakho…*

Uvavanyo	Impendulo efunekayo	Elungileyo/Engalunganga
1. ngcamla	1	
2. mtyibilizi	3	
3. umngxumo	1	
4. ephothiweyo	4	
5. khuselekile	1	
6. ntupha	1	
7. khatha	2	
8. fukufuku	1	
9. ichibi	1	
10. ihlathi	1	
11. zimela	3	
12. acwengileyo	2	
13. ulwamvila	2	
14. ngcileza	2	
15. krekretha	2	
**Isambuku sempendulo ezichaniweyo**		
